# Long-term outcomes for epidemic viral pneumonia survivors after discharge from the intensive care unit: a systematic review

**DOI:** 10.31744/einstein_journal/2024RW0352

**Published:** 2024-02-29

**Authors:** Larrie Rabelo Laporte, Alexandre von Flach Garcia Chavez, Otavio Tavares Ranzani, Juliana Caldas, Rogerio da Hora Passos, João Gabriel Rosa Ramos

**Affiliations:** 1 Clínica Florence Salvador BA Brazil Clínica Florence, Salvador, BA, Brazil.; 2 Escola Bahiana de Medicina e Saúde Pública Salvador BA Brazil Escola Bahiana de Medicina e Saúde Pública, Salvador, BA, Brazil.; 3 Barcelona Institute for Global Health Universitat Pompeu Fabra Centro de Investigación Biomédica en Red de Epidemiología y Salud Pública Barcelona Spain Barcelona Institute for Global Health, Universitat Pompeu Fabra; Centro de Investigación Biomédica en Red de Epidemiología y Salud Pública, Barcelona, Spain.; 4 Hospital das Clínicas Faculdade de Medicina Universidade de São Paulo São Paulo SP Brazil Pulmonary Division, Instituto do Coração (InCor), Hospital das Clínicas, Faculdade de Medicina, Universidade de São Paulo, São Paulo, SP, Brazil.; 5 Intensive Care Unit Hospital São Rafael Salvador BA Brazil Intensive Care Unit, Hospital São Rafael, Salvador, BA, Brazil.; 6 Instituto D’Or de Ensino e Pesquisa Salvador BA Brazil Instituto D’Or de Ensino e Pesquisa, Salvador, BA, Brazil.; 7 Internal Medicine Department Universidade Federal da Bahia Salvador BA Brazil Internal Medicine Department, Universidade Federal da Bahia, Salvador, BA, Brazil.

**Keywords:** Respiratory distress syndrome, Middle east respiratory syndrome coronavirus, Coronavirus infections, Treatment outcomes, Influenza a virus, H1N1 subtype, Epidemics, Intensive care units, Quality of life, Pneumonia, viral, Return to work, Mortality

## Abstract

**Objective:**

To review the long-term outcomes (functional status and psychological sequelae) of survivors of critical illnesses due to epidemic viral pneumonia before the COVID-19 pandemic and to establish a benchmark for comparison of the COVID-19 long-term outcomes.

**Methods:**

This systematic review of clinical studies reported the long-term outcomes in adults admitted to intensive care units who were diagnosed with viral epidemic pneumonia. An electronic search was performed using databases: MEDLINE^®^, Web of Science™, LILACS/IBECS, and EMBASE. Additionally, complementary searches were conducted on the reference lists of eligible studies. The quality of the studies was assessed using the Newcastle-Ottawa Scale. The results were grouped into tables and textual descriptions.

**Results:**

The final analysis included 15 studies from a total of 243 studies. This review included 771 patients with Influenza A, Middle East Respiratory Syndrome, and Severe Acute Respiratory Syndrome. It analyzed the quality of life, functionality, lung function, mortality, rate of return to work, rehospitalization, and psychiatric symptoms. The follow-up periods ranged from 1 to 144 months. We found that the quality of life, functional capacity, and pulmonary function were below expected standards.

**Conclusion:**

This review revealed great heterogeneity between studies attributed to different scales, follow-up time points, and methodologies. However, this systematic review identified negative long-term effects on patient outcomes. Given the possibility of future pandemics, it is essential to identify the long-term effects of viral pneumonia outbreaks. This review was not funded. Prospero database registration: (www.crd.york.ac.uk/prospero) under registration ID CRD42021190296.

## INTRODUCTION

Major epidemic events related to viral pneumonia have occurred in the last decades. A few of these qualify as pandemics, such as Severe Acute Respiratory Syndrome (SARS), Middle East Respiratory Syndrome (MERS), Influenza A (H^1^N^1^), and, most recently, coronavirus disease (COVID-19).^(1-4)^This increase in the rate of emergent respiratory viral infections is attributed to several factors such as growth in the human population, urbanization, changes in the interactions between human and animal populations, variations in climate, and increases in international travel and trade.^(5)^

These epidemics have been associated with high mortality and massive hospitalizations, especially in intensive care units (ICUs), leading to an overload of the health system.^(6-8)^In 2003, the SARS outbreak exposed significant global weaknesses in coping with the newly emerged viral pneumonia epidemic.^(9)^ Approximately 20 years later, the world faced a similar challenge with the emergence of COVID-19, which led to nearly six million deaths worldwide from December 2019 to February 2022.^(10)^

Along with acute challenges, these viral pneumonia outbreaks have resulted in significant long-term clinical challenges for survivors, with implications for rehabilitation services and healthcare utilization.^(11)^ Currently, follow-up studies of COVID-19 have demonstrated significant long-term sequelae, which have been named “long COVID.” However, uncertainty exists regarding whether the post-COVID-19 burden is due to the impact of acute illness, which is worsened by a stressed healthcare system, or intrinsic characteristics of SARS-CoV-2.^(12,13)^

Therefore, we systematically reviewed the long-term outcomes (functional status and psychological sequelae) of survivors of critical illnesses due to epidemic viral pneumonia (SARS, MERS, and H^1^N^1^) before COVID-19 to establish a benchmark for comparison with COVID-19 long-term outcomes and help to establish metrics for preparation for possible upcoming pandemics.

## OBJECTIVE

To review the long-term outcomes (functional status and psychological sequelae) of survivors of critical illnesses attributable to epidemic viral pneumonia (SARS, MERS, and H^1^N^1^) before COVID-19 to establish a benchmark for the comparison with long-term outcomes of COVID-19.

## METHODS

This study adhered to the principles of the Preferred Reporting Items for Systematic Reviews and Meta-Analyses (PRISMA) consensus.^(14)^

### Literature search

Articles were searched in the following databases: MEDLINE^®^, Web of Science, LILACS/IBECS (*Biblioteca Virtual em Saúde* (BVS]), and EMBASE. The time limit of the bibliographic search ranged from inception to July 2020. Furthermore, an additional investigation was conducted on the reference lists of the eligible studies. Appendix 1 presents a search strategy using Boolean descriptors and operators. The keywords were matched in English and Portuguese and registered in the National Library of Medicine’s controlled vocabulary thesaurus (MeSH).

**Appendix 1:** Search strategy for the systematic review

Database	Keywords	Results
Pubmed	((MERS[All Fields] OR “Middle East respiratory syndrome”[All Fields] OR SARS[All Fields] OR “severe acute respiratory syndrome”[All Fields] OR coronavirus[All Fields] OR H1N1[All Fields] OR influenza[All Fields] OR “viral pneumonia” [All Fields] OR pandemics[All Fields]) OR (MERS[MeSH Terms] OR “Middle East respiratory syndrome coronavirus” [MeSH Terms] OR “SARS virus”[MeSH Terms] OR “severe acute respiratory syndrome”[MeSH Terms] OR “Coronavirus infections”[MeSH Terms] OR “Influenza A Virus, H1N1 Subtype”[MeSH Terms] OR “Influenza, Human”[MeSH Terms] OR “Pneumonia, Viral”[MeSH Terms] OR pandemics[MeSH Terms])) AND ((ICU[All Fields] OR “intensive care unit”[All Fields] OR “critical care unit”[All Fields] OR “critical care”[All Fields] OR “critically ill”[All Fields] OR “critical illness”[All Fields]) OR (“intensive care units”[MeSH Terms] OR “critical care”[MeSH Terms] OR “critical illness”[MeSH Terms])) AND (((((((((“outcome measure”[All Fields] OR “long term outcomes”[All Fields]) OR “quality of life”[All Fields]) OR “functional status”[All Fields]) OR “recovery of function”[All Fields]) OR “recovery of function”[MeSH Terms]) OR “follow up studies”[All Fields]) OR “muscle strength”[All Fields]) OR “posttraumatic stress disorder”[All Fields]) OR “employment”[All Fields])	190
EMBASE / Web of Science / LILACS	(MERS OR “Middle East respiratory syndrome” OR SARS OR “severe acute respiratory syndrome” OR coronavirus OR H1N1 OR influenza OR “viral pneumonia” OR pandemics) AND (ICU OR “intensive care unit” OR “critical care unit” OR “critical care” OR “critically ill” OR “critical illness”) AND ((“outcome measure” OR “long term outcomes”) OR “quality of life” OR “functional status” OR “recovery of function” OR “follow up studies” OR “muscle strength” OR “posttraumatic stress disorder” OR “employment”)	50

### Data collection and selection of studies

Two researchers independently performed the study identification, selection, and data collection. Duplicate records were automatically excluded using the Mendeley Desktop tool (version 19.1.4). In cases of discordance, a third senior researcher provided a definitive answer. First, titles and abstracts were evaluated for the initial selection of the identified articles. Second, the full articles were read and those fulfilling the inclusion criteria were included in the data collection phase for qualitative analysis. Third, the data on the outcomes of interest in the selected articles were collected.

### Inclusion and exclusion criteria

Studies published in English, Spanish, French, and Portuguese were included as follows: participants older than 18 years who survived intensive care unit admission due to viral pneumonia during an epidemic period; participants whose long-term outcomes were investigated after ICU discharge; and patients with a diagnosis of viral pneumonia (SARS, MERS, H^1^N^1^).

The exclusion criteria were as follows: manuscripts that did not have an abstract or full text; studies that described only the long-term serological status or radiological findings of the diseases under consideration; studies addressing patients aged <18 years; studies without follow-up of the patient after discharge from the hospital; and studies including animals and review studies.

### Evaluated outcomes

Long-term outcomes (quality of life, function, survival, psychological measures, and employment) and resource utilization (rehospitalization and long-term acute care facility utilization) were evaluated.

We could not access the subgroup analysis of viral agents and age subgroups (<60; 60-80; >80 years) as planned in the protocol owing to the lack of individual information regarding outcomes by age group in each study.

### Methodological quality assessment

The quality of the articles was independently assessed by two authors based on the items proposed by the Newcastle-Ottawa Scale (NOS).^(15)^ Overall confidence was scored from 0 to 9 stars. The greater the number of stars, the better the methodological quality. We did not assess the study quality using the CLARITY Group from McMaster University as proposed in the protocol because of Cochrane’s recommendation and the familiarity of authors in applying and interpreting the NOS Scale.

### Data extraction and analysis

Articles were organized and read using the Mendeley Desktop tool (version 19.1.4). The following data were collected from the selected papers: study identification (title, author(s), year of publication, country of origin, scientific journal of publication, language, and keywords), objective(s); study design; methods; inclusion and exclusion criteria; sociodemographic aspects (age and sex of participants); viral etiology of pneumonia; length of follow-up; and instruments used for measuring functional status, quality of life, and mental health status. Additionally, we collected the following outcome variables: length of ICU stay; post-discharge mortality rate; functional status during follow-up; quality-of-life during follow-up; percentage of forced expiratory volume in one second (FEV^1^); percentage of the level of diffusion capacity of carbon monoxide (LDCO); rate of return to work; and recharge.

A meta-analysis could not be performed due to the extensive variation in the types of scales, variables, and outcomes assessed. Therefore, we conducted a systematic descriptive study using tables and textual descriptions.

## RESULTS

### Selection of studies

**Figure 1:**
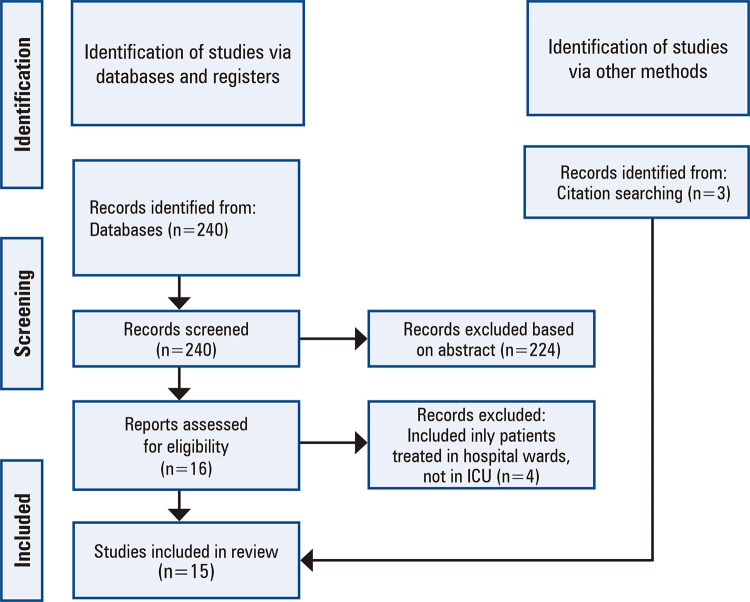
Flowchart for selection of studies

The initial search yielded 240 articles from the described databases. An additional three studies discovered through previous external searches were included. Among them, 19 were selected for full-text reading. After each article was read, 15 studies were included in the review. A flowchart of all the selection processes is shown in figure 1.

### Study characteristics

This review included patients with H1N1,^(16-23)^MERS,^(24)^and SARS.^(24-29)^ Further, it analyzed mortality,^(16,22,23,26)^ rehospitalization,^(20)^quality of life,^(17-19,21,24-27,29,30)^ lung function,^(17-21,26-29)^ functionality,^(17,18,20,21,26,27,29)^ return to work,^(20,21,24,25,27)^ and psychiatric illnesses.^(17,21)^ Follow-up duration ranged from 1^(18)^ to 144 months.^(25)^ The sample sizes ranged from 4^(20)^ to 135^(24)^ participants, resulting in 771 individuals with a mean age of 29.9 years and a male proportion ranging from 43%^(25)^ to 100%.^(18,20)^ All the patients included in the analyses were admitted to the ICU during their hospital stay.

All 15 studies were conducted after the year 2000. Eight studies were from Asia^(18,23-29)^ and only three were multicenter studies.^(21,24,30)^ One study was interventional,^(25)^ whereas the others were observational,^(16-24,26-30)^ prospective,^(18,21-23,25,27-30)^ retrospective,^(16,26)^ cross-sectional,^(24)^ or case series.^(17,19,20)^ Four studies included control groups for outcome comparison. Guo et al.,^(25)^ Luyt et al,^(21)^ and Quispe-Laime et al.^(19)^ compared patients with viral pneumonia treated with or without oseltamivir; acute respiratory distress syndrome (ARDS) due to H1N1 treated with or without extracorporeal lung assist (ECLA) devices; and ARDS due to or not due to H1N1, respectively. The methodological quality of the included studies ranged from 5-9 stars. Table 1 presents descriptive information of the included studies.

**Table 1:** Description of included studies

Author	Year	Country	Study Design	Condition	Follow up, months	Sample	Groups	Age	Males %	ICU length of stay	Outcome	NOS
Bal at al.^(16)^	2020	France	Retrospective	H1N1	3	45	-	56.7±15.6	60	23.3 (8.0 –32.0)	Mortality	6
Biswas et al.^(17)^	2016	Ireland	Case Series	H1N1	12	7	-	44.7±13.7	-	-	Quality of life Functional capacity Pulmonary function Psychiatric disorders	5
Hsieh et al.^(18)^	2018	Taiwan	Prospective	H1N1	1, 3 and 6	9	-	45.11±5.48	100	16.89±2.51	Quality of life Functional capacity Pulmonary function	6
Quispe-Laime et al.^(19)^	2012	Argentina	Case Series	H1N1	6	11	-	37.27±9.4	72.7	13.73±7.016	Quality of life Pulmonary function	6
Toufen et al.^(20)^	2011	Brazil	Case Series	H1N1	2 and 6	4	-	37.5±13.5	100	19.5±5.8	Rehospitalization Back to work Pulmonary function	6
Luyt et al.^(21)^	2012	France	Prospective	H1N1	12	37	ECLA	35.5 (30– 39)	42	37.5 (19–67)	Back to work Quality of life Functional capacity Pulmonary function Psychiatric disorders	8
							Control	42 (32.75– 51.25)	52	19 (12–27.5)		
Fariña et al.^(22)^	2009	Argentina	Prospective	H1N1	1	5	-	34 (23–54)	40	-	Mortality	5
Hou et al.^(23)^	2012	China	Prospective	H1N1	1 and 3	9	-	31.2 (21–59)	31.9	31.2 (21–59)	Mortality	5
Batawi et al.^(24)^	2019	Saudi Arabia	Transversal	MERS + SARS	13.8	135	MERS	44.99±12.95	71.8	-	Back to work Quality of life	8
							Non-MERS	50.04±13.64	59.6			
Guo et al.^(25)^	2019	China	Prospective	SARS	144	67	Oseltamivir	29.91±10.11	32	24.03±8.40	Back to work Quality of life	8
							Placebo	36.97±13.24	48	16.26±7.24		
Li et al.^(26)^	2006	Hong Kong	Retrospective	SARS	3, 6 and 12	59	-	47±15.7	58	9 (5–20)	Mortality Quality of life Functional capacity Pulmonary function	9
Ngai et al.^(27)^	2010	Hong Kong	Prospective	SARS	3, 6, 12, 18 and 24	123	-	33.4±8.6	41.8	-	Back to work Quality of life Functional capacity Pulmonary function	9
Yin et al.^(28)^	2005	China	Prospective	SARS	1, 3 and 6	93	-	39.8±13.8	31.9	-	Pulmonary function	5
Hui et al.^(29)^	2005	Hong Kong	Prospective	SARS	1, 3 and 6	110	-	35.6±9.8	40	13.5±15.6	Quality of life Functional capacity Pulmonary function	8
Skinner et al.^(30)^	2015	Australia	Prospective	H1N1	12	62	-	42 (29–53)	48	20.0 (15–38)	Quality of life	7

ARDS: acute respiratory distress syndrome; SARS: severe acute respiratory syndrome; MERS: the Middle East respiratory syndrome; DLCO: diffusing capacity for carbon monoxide; 6 MWT: 6-minute walk test; ICU: intensive care unit; FEV1: forced expiratory volume in 1 seconds; ECLA: extracorporeal lung assist; MRC: medical research council; NOS: Newcastle–Ottawa Scale.

### Functional capacity

Functional capacity was evaluated in seven studies, with six using the 6-minute walk test (6 MWT) scale^(17,18,20,26,27,29)^ and one using the Medical Research Council (MRC) scale.^(21)^ Hsieh et al.^(18)^ reported a significant improvement in the 6 MWT from 1 to 3 months after hospital discharge, with further improvement from 3 to 6 months. Li et al.^(26)^ and Ngai et al.^(27)^ reported improvement from 3 to 6 months; however, no significant change was observed at 12 months in the 6 MWT. Two small case series by Biswas et al.^(17)^ and Toufen et al.^(20)^ reported varying levels of improvement in the 6 MWT, with a few patients experiencing physical limitations and significant desaturation during testing. Luyt et al.^(21)^ evaluated muscle strength using the MRC scale and observed similar near-normal test results in both the ECLA and non-ECLA groups. Table 2 reveals additional information regarding the 6 MWT.

**Table 2:** Assessment of the functional capacity using the 6-minute walk test

Author	Year	Condition	Time, months	6MWD (m) [Mean (SD or IQR)]
Biswas et al.^(17)^	2016	H1N1	12	500 (94)
Hsieh et al.^(18)^	2018	H1N1	1	486.6 (150–682)
			3	551 (470–625)
			6	604.8 (482–716)
Toufen et al.^(20)^	2011	H1N1	6	501 (65.38)
Li et al.^(26)^	2006	SARS	3	454 (98)
			6	504 (107)
			12	506 (111)
Ngai et al.^(27)^	2010	SARS	3	439.0 (89.1)
			6	460.1 (102.8)
			12	464.7 (101.9)
			18	466.3 (91)
			24	462.6 (120)
Hui et al.^(29)^	2005	SARS	3	464 (87)
			6	502 (97)
			12	511 (90)

SARS: severe acute respiratory syndrome.

### Return to work

The return to work rates of patients was assessed in five studies^(20,21,24,25,27)^ with follow-up periods ranging from 6 to 144 months. According to Luyt et al.^(21)^ and Toufen et al,^(20)^ approximately 70% of patients return to normal activities at 12 and 6 months, respectively. Batawi et al.^(24)^ and Ngai et al.^(27)^ stated that approximately 80% of individuals returned to their pre-hospitalization work function 12 months after discharge, with no significant difference between MERS and non-MERS SARS survivors as reported by Guo et al.^(25)^ followed patients for 12 years and noted that 92% returned to work.

### Quality of life

Ten of the fourteen studies evaluated the quality of life of patients using different questionnaires, such as the Short Form 36 (SF-36),^(21,24-27,29,30)^St. George’s Respiratory Questionnaire (SGRQ),^(18)^EuroQol- 5 Dimension (EQ-5D),^(19)^and General Health Questionnaire (GHQ).^(17)^ For example, Batawi et al.^(24)^ observed that SF-36 physical component scores were significantly lower in MERS-CoV survivors admitted to the ICU than in those managed in a medical ward after 1 year of follow-up. In addition, Guo et al.^(25)^ and Luyt et al.^(21)^ demonstrated that both the physical and mental component scores of the SF-36 were below average for a healthy population at 12 years and 12 months after discharge from the ICU, respectively.

Skinner et al.^(30)^ reported physical and mental component scores in the SF-36 to be within the normal range in the population at 12 months. Additionally, Hui et al.^(29)^ stated no significant difference in SF-36 domains between age groups at 6 months, except for a subgroup analysis with a lower score in bodily pain for patients aged 41-64 who required ICU support.

Quispe-Laime et al.^(19)^ evaluated patients using the EQ-5D and observed a mean score of 70%, with changes in all five components at 6 months after discharge from the ICU. Hsieh et al.^(18)^ used the SGRQ and showed improvement in questionnaire values over 6 months, reaching the expected values of a healthy population. Biswas et al.^(17)^ reported the values expected for a healthy population using the GHQ 12 months after discharge from the ICU. Furthermore, Li et al.^(26)^ and Ngai et al.^(27)^ evaluated the quality of life; however, they did not provide sufficient descriptive data for comparison. The results are summarized in table 3.

**Table 3:** Description of follow-up health-related quality of life

Scale	Author	Year	Condition	Time, months	Group	Quality of life [Mean (SD or IQR)]
GHQ	Biswas et al.^(17)^	2016	H1N1	12	-	16 (2)
SGRQ	Hsieh et al.^(18)^	2018	H1N1	1	-	29.4
				6	-	4.8
EQ-5D	Quispe-Laime et al.^(19)^	2012	H1N1	6	-	70 (24.5)
SF-36	Luyt et al.^(21)^	2012	H1N1	12	ECLA	PCS: 47.1 (5.6)
						MCS: 44.0 (12.0)
					Control	PCS: 44.7 (10.9)
						MCS: 43.3 (11.2)
	Batawi et al^(24)^	2019	MERS	12	-	PCS: 64.84 (25.52)
					-	MCS: 74.82 (25.14)
	Guo et al.^(25)^	2019	SARS	144	Oseltamivir	PCS: 68.33 (10.3)
						MCS: 73.28 (12.42)
					Control	PCS: 76.28 (14.06)
						MCS: 76.41 (9.22)
	Hui et al.^(29)^	2005	SARS	6	18–40 years	PCS: 96.3 (6.6)
						MCS: 71.8 (15.6)
					41–64 years	PCS: 90.6 (12.4)
						MCS: 73.1 (17.6)
	Skinner et al.^(30)^	2015	H1N1	12	-	PCS: 44.4 (12.3)
						MCS: 45.5 (12.5)

SARS: severe acute respiratory syndrome; MERS: Middle East respiratory syndrome; ECLA: extracorporeal lung assist; PCS: physical component summary; MCS: mental component summary.

### Pulmonary function

Eight studies^(17-21,26-29)^ investigated the variations in diffusing capacity for carbon monoxide (DLCO) and FEV1 among patients with epidemic viral pneumonia from 1 to 24 months after ICU discharge. Hsieh et al.^(18)^ observed significant improvements in DLCO and FEV1 3 months after hospital discharge compared with that at 1 month; however, there were no improvements when comparing DLCO and FEV1 at 3 and 6 months. Nevertheless, they exhibited lower values compared with the healthy population. The studies by Quispe-Laime et al.^(19)^ and Biswas et al.^(17)^ indicated that the FEV1 and DLCO reflected normal lung function during the entire follow-up, and Yin et al.^(28)^ found that DLCO normalized 5 months after discharge.

Li et al.^(26)^ and Guo et al.^(25)^ stated that DLCO at 12 and 144 months was significantly higher in patients who did not require mechanical ventilation than in those who did. Moreover, Luyt et al.^(21)^ established that the ECLA and no-ECLA groups (75% and 64%, respectively) had decreased but had comparable DLCO levels despite their near-normal and similar lung function test results. Additionally, Hui et al.^(29)^ observed a negative correlation between DLCO and radiographic abnormalities but not with FEV1.

### Mortality

Mortality in patients with viral epidemic pneumonia was reported in four studies.^(16,22,23,26)^ Li et al.^(26)^ and Bal et al.^(16)^ did not report any post-discharge mortality in the ICU. However, the total mortality during the ICU stay, hospitalization, and follow-up period was 24% at 12 months and 33% after 3 months, respectively. In contrast, Hou et al.^(23)^ reported a higher mortality rate of 44.4% at 3 months after discharge. Finally, Fariña et al.^(22)^ stated that one out of five patients died 30 days after discharge.

### Psychiatric disorders

Two studies^(16,20)^ investigated the presence of psychiatric disorders in patients with viral epidemic pneumonia. Biswas et al.^(17)^ utilized the Beck Anxiety and Depression Inventory and stated an average anxiety score of 2.1 and a depression score of 3.7, which are typical values for a healthy population. Luyt et al.^(21)^ assessed the impact of event scale-revised, compared patients who received ECLA, and observed similar rates of depression (28% *versus* 28%), anxiety (50% *versus* 56%), and risk for PTSD (41% *versus* 44%).

### Rehospitalization

In the study by Toufen et al,^(20)^ one patient (25% of the sample) was readmitted twice to the hospital because of pulmonary edema.

## DISCUSSION

Our findings suggest that before COVID-19, survivors of pandemic viral pneumonia experienced worsening quality of life, mental disorders, deterioration of functional capacity, and worsening of pulmonary function after discharge from the ICU. This is the first systematic review to address the long-term outcomes of critically ill survivors of non-COVID epidemic pneumonia, including SARS, MERS, and H1N1 pneumonia.

The quality of life is a critical issue for survivors of acute illnesses. Our study revealed that the quality of life is reduced in viral pneumonia survivors. Although most studies used the SF-36 to evaluate the quality of life, there was significant heterogeneity in the measurement tools, comprising different scales, time points, and methodologies, such that we observed discrepant results. For example, in studies that assessed SF-36, the physical and mental scores of patients with viral pneumonia were lower than the average population, with a few studies indicating no improvement after 1 or 12 years of follow-up. However, it improved over time in studies that used other scales including the SGRQ, EQ-5D, and GHQ, leading to 6-12-month values such as those observed in a healthy population.^(18)^ This may be attributed to differences in the application and evaluation of each domain in the different questionnaires.

This review revealed that survivors may have psychological conditions such as PTSD, depression, and anxiety beyond 12 months. The high risk of post-traumatic stress disorder and long-term depressive and anxiety symptoms observed in this review is comparable to that in patients with ARDS^(31)^ with a high prevalence of depression and anxiety in general intensive care unit survivors.

Regarding functional capacity, studies using the 6 MWT in this review reported a significant improvement at 1, 3, and 6 months, with a slight, however, not significant improvement at 12 months after discharge. Despite progress in the first 6 months, the percentage values remained below the population average. This improvement trend reached a plateau at 12 months, although it remained below the average of a healthy control population, which was observed in one meta-analysis by Ahmed et al. that addressed long-term outcomes of coronavirus outbreaks after hospitalization, but not necessarily after admission to the ICU.^(32)^ Other systematic reviews have reported a similar decline in functionality in the general population of critically ill patients, such as the review conducted by Ohtake et al., who evaluated post-intensive care syndrome.^(33)^

The included studies reported a return to work rate of up to 70% until 1 year after discharge, similar to a German cohort that demonstrated a lower rate of 64% at 5 years in patients with ARDS after ICU discharge.^(34)^ In addition, Luyt et al.^(21)^ discovered a higher return to work rate of 83% in H1N1 patients who received ECMO compared with 64% of those who did not, which suggests that disease severity may not be the only factor affecting long-term outcomes. In contrast, Wilcox et al. reported a lower rate of approximately 50% of patients with general ARDS who required ECMO one year after discharge.^(35)^

Regarding pulmonary function in patients with viral pneumonia admitted to the ICU, the DLCO and FEV1 values after 6 months were similar to those in healthy individuals. The most significant improvement in these parameters occurred during the first 3 months after discharge. Mechanical ventilation was a significant predictor of poor pulmonary function in patients who did not require mechanical ventilation and had better lung function test results. This difference may be explained by the poor prognosis of patients requiring mechanical ventilation.^(36)^

This study has few limitations. Different scales were used to evaluate the outcomes, which resulted in significant heterogeneity. This affected the data appraisal and posed a challenge in summarizing the results. Therefore, this review highlights the need to establish data collection standards in follow-up studies of critically ill patients. It is crucial to improve the quality of data collection to enhance the robustness and generalizability of future studies in this area. Another limitation is that incomplete population characteristics in specific studies, specifically separating ICU and non-ICU patients, may have led to the exclusion of studies that did not fit the research question. Additionally, two studies were excluded from the quality of life analysis because they did not provide data or a descriptive analysis of the outcome and only stated whether it had statistical significance.

This systematic review addresses the importance of identifying the outcomes and impact of pandemic/epidemic viral pneumonia outbreaks, given the possibility of future episodes. However, it is challenging to compare the effect of this viral pneumonia with other severe pneumonia cases, as they may be influenced not only by the disease itself but also by the strain on healthcare systems owing to the number of patients simultaneously impacted. To address this gap, this novel study analyzed a wide range of patients with epidemic viral pneumonia that required ICU admission and evaluated their long-term outcomes, going beyond previous studies that only focused on COVID-19,^(37)^ included only other viral pandemics, or included non-critically ill patients.^(32)^

This review has several strengths. First, it followed the PRISMA guideline and searched multiple databases in four languages, ensuring comprehensive coverage of available evidence. Moreover, the study evaluated several critical long-term outcomes by comprehensively analyzing the perceptions and results of those affected by the disease. These strengths render this review a valuable resource for health systems, researchers, and policymakers seeking to understand and mitigate the long-term impacts of epidemic viral pneumonia, establish a baseline for comparison of COVID-19 results, and improve preparedness for hypothetical upcoming pandemics.

## CONCLUSION

This systematic review demonstrated a reduction in the quality of life, functionality, and expected percentage of diffusing capacity for carbon monoxide in patients with viral epidemic pneumonia after discharge from the intensive care unit. The data presented may be compared with those of studies analyzing the long-term outcomes of COVID-19 patients. However, additional studies using homogeneous evaluation methods are necessary to obtain more reliable results and their possible extrapolations to new outbreaks.
